# Reference values of electrographic and cardiac ultrasound parameters in Russian healthy children and adolescents

**DOI:** 10.1038/s41598-021-82314-0

**Published:** 2021-02-03

**Authors:** Geraldine Landon, Isabelle Denjoy, Enora Clero, Aleksandr Silenok, Irina Kurnosova, Andrey Butsenin, Patrick Gourmelon, Jean-Rene Jourdain

**Affiliations:** 1 Division of Health, Institute for Radiological Protection and Nuclear Safety (IRSN), BP 17, 92262 Fontenay-aux-Roses cedex, France; 2 Department of Cardiology, Bichat Hospital (AP-HP), Paris, France; 3 Department of Cardiology, Bryansk Diagnostic Center, Bryansk, Russia; 4 European and International Affairs Department, Institute for Radiological Protection and Nuclear Safety (IRSN), BP 17, 92262 Fontenay-aux-Roses cedex, France

**Keywords:** Cardiology, Medical research

## Abstract

Between 2009 and 2013, a large cross-sectional study on the health consequences of the Chernobyl nuclear accident was performed in the contaminated and uncontaminated territories of the Bryansk Oblast (Russian Federation). The objective of this work was to confirm or refute a possible association between childhood cardiac arrhythmia and a chronic exposure to caesium-137. As part of this study, a large number of electrocardiographic and cardiac ultrasound parameters were collected from 18,152 children aged 2–18 years including 12,512 healthy ones not contaminated with caesium-137. It seemed therefore relevant for us to share in a second publication these medical data based on healthy and uncontaminated children with the scientific community because of the large quantities and the limited availability of such kind of data. In the present study, relating to electrocardiographic parameters, the measurements performed fully reflect the expected evolution of the paediatric electrocardiogram between 5 and 18 years of age. Thus, the median values were generally quite close to those available in the literature. In contrast, differences in the 2nd and 98th percentiles were notable and could be explained in particular by the type of equipment used, the number of subjects included in the study and racial disparities. As for echocardiographic parameters, the evolution of the measured values in age groups is consistent with what was expected considering factors such as growth. In comparison with other scientific studies that have investigated these echocardiographic parameters, some differences by age groups have been identified. The ethnic factor truly appears to be a relevant feature to consider. In view of the results, it appeared essential to the authors to approach the methodological conditions of the scientific studies already published on the topic to be truly comparable and thus to provide a reliable answer on a topic for which real expectations in terms of medical care are required.

## Introduction

In the aftermath of the Chernobyl nuclear accident, many questions have arisen and remain relating to non-cancer effects resulting from chronic exposure to low doses of ionizing radiation. In light of this context, the French Institute for Radiological Protection and Nuclear Safety (IRSN) has decided to implement a large-scale cross-sectional population-based study to explore the possible association between cardiac arrhythmias, a pathology observed on the rise in contaminated territories, and caesium-137, a radionuclide found mostly in the radioactive plume and which is still considered as the reference radionuclide to assess the ground contamination^1,2^. As the only health effect undoubtedly attributable to the accident is the thyroid cancer in children, we decided to focus our attention on this sensitive population^2,3^. All of the results of this four-year study (2009–2013), which was conducted on the contaminated and uncontaminated territories of Bryansk *Oblast* (Russian Federation) were published in 2018^1^. As part of comprehensive cardiac assessment, an electrocardiogram (ECG) and a cardiac ultrasound were obtained on the subjects. Currently, no harmonized reference values for ECG and echocardiographic parameters in children are available^4,5^. Having these reference values thus appears crucial to ensure the relevant medical care of young patients^6,7^. In order to be useful and comparable, for each medical parameter and according to age group and gender, we assessed the median as well as the 2nd and 98th percentiles considered in the medical field as the limit values^8,9^.

Throughout our study, given the numerous medical data collected, the present article aims to compare our results with those already published for encouraging the debate on the topic of the reference values of electrographic and cardiac ultrasound parameters and finally to support or not the conclusions drawn from studies previously conducted.

## Methods

### Study population

The data come from a cross-sectional study conducted in south-western region of Bryansk (Russian Federation) on 18,152 children aged 2–18 years at time of medical examination^1^. From May 2009 to May 2013, field medical teams gathering health data in schools were instructed to explain beforehand the major objectives of the study and the different medical examinations planned. A written informed consent was obtained from the parents. No financial remuneration was granted to the individuals. Among the examined children, 455 (2.5%) were excluded because of either cardiac arrhythmia associated with a congenital pathology or other cardiac pathology. To investigate the reference values of ECG and echocardiographic parameters, the present work only focuses on participants not presenting cardiac disorders and whole-body caesium-137 contamination *i.e.* 12,512 children.

The study protocol complied with the guidelines laid down in the Declaration of Helsinki and was approved and validated by the Ethics Committee of the Russia’s National Hematology Center in Moscow (number of ethical approval: 58).

### Medical examinations

Before conducting medical examinations, the medical team collected socio-demographic data (gender, age, place of residence), medical history, information relating to the medication intake that may lead to cardiac arrhythmia within the month prior to medical examinations, and anthropometric parameters such as height, weight, body mass index (BMI) and body surface area (BSA). BMI was defined as weight (in kg) divided by squared height (in m) and the percentiles specific for age and gender were used to categorize the children according to the World Health Organization (WHO) classification (underweight, normal weight, overweight and obesity). BSA (in m^2^) was obtained using Dubois & Dubois formula^10^ hereafter:

$$BSA=0.007184 \times {H}^{0.725 }\times {W}^{0.425}$$

where W is the weight (in kg) and H is the height (in cm).

For practical reasons and for the well-being of the children, it was decided to collect data in schools, which avoided to disturb as little as possible their daily routines. Once these data registered, the study population underwent medical examinations, including an ECG and an echocardiography. All the medical information was compiled into a health questionnaire and then recorded in a digital format into a database (Access).

### Electrocardiographic parameters

The ECG which is the first-line diagnostic tool indivisible from any cardiovascular clinical examination represents the trace of electrical activity of the heart. Through the analysis of the different phases of the cardiac cycle, the physician ensures the proper functioning of the organ or, on the contrary, detects cardiac diseases such as arrhythmia. The analysis primarily focuses on the following elements: P wave referring to the auricular depolarization/(or systole); PR interval following the auriculo-ventricular conduction; the ventricular depolarization which is studied through QRS complex and finally, T wave corresponding to the repolarization of the ventricles (or diastole) and QT interval which is associated to the ventricular systole period. The measurements of electrocardiographic parameters were performed using Cardiosoft version 6.5 diagnostic system (GE Medical Systems Information Technologies GmbH Inc., Freiburg, Germany) which acquired and analyzed resting ECG with Marquette ECG analysis programs. The simultaneous 12-lead ECG was recorded after a 10-min rest, in the supine position, at a sampling rate of 4 kHz (with a bandwidth of 150 Hz and a standard paper speed of 25 mm/s). The following ECG parameters were evaluated: heart rate (bpm), PR interval (ms), P axis (°), P wave (ms), QRS complex (ms), QT interval (ms), corrected QT interval (QTc) (ms), RR interval (ms), R axis (°) and T axis (°). Regarding the QTc, it was calculated according to Bazett’s formula below^11^.

$$QTc = \frac{{QT}}{{\sqrt {\frac{{60}}{{HR}}} }}$$

where HR corresponds to heart rate (bpm).

All ECG tracings were recorded and available in the database.

### Echocardiographic parameters

To carry out the transthoracic Doppler echocardiography, an ultrasound diagnostic scanner MyLab 30 CV (ESAOTE, Genova, Italy) equipped with a phased array probe PA230E P.A. 2–4 MHz was used. B and M modes were employed. From this examination the following parameters were assessed: the left ventricular diameter at end-systole (LVEDs, in mm), left ventricular diameter at end-diastole (LVEDd, in mm), left ventricle ejection fraction (LVEF, in %), left ventricular posterior wall thickness at end-diastole (LVPWd, in mm), left ventricle velocity (in cm/sec), interventricular septum thickness at end-diastole (IVSd, in mm), right ventricular diameter at end-diastole (RVDd, in mm), mitral inflow velocity (in cm/sec), left atrial diameter (LAD, in mm), aortic diameter (AOD, in mm), tricuspid peak velocity (in cm/sec), trunk diameter of the pulmonary artery (in mm), and pulmonary artery velocity (in cm/sec).

To overcome a potential "dependent operator" bias, electrocardiography and echocardiography were carried out by a single paediatric cardiologist who always took the necessary time to document his diagnosis^12,13^.

### Statistical analysis

Statistical analyses were performed with SAS software version 9.2 (SAS Institute, Inc., Cary, NC, USA). Analyses of ECG and echocardiographic parameters were conducted by gender and by age group (5–7; 8–11; 12–15; 16–18 years). The results were expressed as median and 2nd and 98th percentiles. Moreover, as it is known that most echocardiographic parameters are strongly correlated with weight and height, we decided to take into account the BSA, parameter the most commonly used in the indexing method^14^.

Statistical tests were two-tailed and p-values were considered statistically significant when below 0.05.

## Results

As already mentioned, to investigate the reference values for ECG and echocardiographic parameters, the analysis was limited to 12,512 healthy children. The baseline characteristics of this Caucasian study population are shown in Table 1.

**Table 1:** Main characteristics of children at time of medical examinations.

Age and anthropometric parameters	Overall *N* = *12,512*		Males *N* = *6145*		Females *N* = *6367*		P-value
**Mean values of children (SD)**							
Age (year)	11.12	(3.23)	11.05	(3.22)	11.20	(3.24)	< 0.01
Height (cm)	146.99	(17.50)	148.02	(18.95)	146.00	(15.92)	< 0.01
Weight (kg)	42.41	(16.13)	43.52	(17.40)	41.34	(14.72)	< 0.01
BMI (kg/m^2^)	18.89	(3.71)	19.04	(3.72)	18.74	(3.69)	< 0.01
BSA (m^2^)	1.32	(0.32)	1.34	(0.34)	1.30	(0.29)	< 0.01
***Number of children (%)***							
**Age group** (year)							
5–7	1973	(15.77)	1004	(8.02)	969	(7.75)	
8–11	4995	(39.92)	2477	(19.80)	2518	(20.12)	
12–15	4050	(32.37)	1960	(15.66)	2090	(16.71)	
16–18	1494	(11.94)	704	(5.63)	790	(6.31)	
**BMI** (kg/m^2^)*							
Underweight	270	(2.16)	107	(0.86)	163	(1.30)	
Normal weight	9173	(73.31)	4298	(34.35)	4875	(38.96)	
Overweight	1774	(14.18)	921	(7.36)	853	(6.82)	
Obesity	1295	(10.35)	819	(6.55)	476	(3.80)	

SD, standard deviation.

Means statistically different between males and females are in bold (p-value < 0.05).

*Based on the WHO classification.

Among the patients, 6145 males and 6367 females participated in the survey, with an overall mean age of 11.12 years. The distribution of children by age group is balanced between males and females, with the “8–11” category being the most represented. In terms of height and weight means, the gap is widening between males and females. Males are on average taller (148.02 *vs* 146.00 cm) and heavier than females (43.52 *vs* 41.34 kg). With respect to the BMI parameter, the distribution of children according to the WHO classification is relatively similar between males and females, with almost three-quarters of children without weight issues. Males seem a little more likely to be overweight or obese than females, 13.91% and 10.62%, respectively. In general, the means are statistically different (p < 0.05), although age and anthropometric parameters are quite close by gender.

### Electrocardiographic parameters

Electrocardiographic recorded parameters are summarized in Table 2 according to age group and gender.

**Table 2:** Electrocardiographic parameters according to age and gender (males, upper row and females, lower row).

Electrocardiographic parameters, median (2nd, 98th percentiles)	Age groups							
	5–7 years N = *1973*		8–11 years N = *4995*		12–15 years N = *4050*		16–18 years N = *1494*	
Heart rate (bpm)	87	(67, 117)	82	(63, 110)	78	(60, 108)	73	(55, 101)
	89	(68, 123)	85	(65, 115)	80	(61, 111)	76	(57, 101)
PR interval (ms)	124	(100, 160)	130	(102, 168)	134	(106, 174)	140	(112, 184)
	124	(98, 158)	128	(100, 164)	134	(104, 172)	136	(112, 176)
P axis (°)	43	(-9, 72)	41	(-10, 71)	45	(-10, 76)	53	(-14, 79)
	45	(-4, 73)	44	(-6, 74)	47	(-5, 76)	50	(-10, 76)
P wave (ms)	82	(66, 100)	86	(66, 104)	90	(72, 108)	94	(74, 114)
	82	(64, 100)	86	(64, 104)	90	(68, 110)	92	(74, 112)
QRS complex (ms)	82	(66, 104)	84	(68, 106)	90	(74, 110)	92	(76, 114)
	78	(64, 98)	80	(66, 102)	84	(70, 106)	86	(70, 106)
QT (ms)	354	(312, 400)	366	(324, 412)	372	(326, 422)	373	(328, 426)
	350	(306, 394)	360	(316, 408)	374	(326, 420)	380	(332, 426)
QTc (ms)	426	(390, 465)	427	(389, 465)	424	(383, 464)	412	(368, 454)
	425	(389, 463)	430	(392, 467)	431	(392, 468)	427	(385, 466)
RR interval (ms)	678	(512, 892)	722	(544, 958)	762	(550, 1004)	820	(594, 1104)
	666	(492, 890)	695	(520, 934)	742	(538, 994)	786	(584, 1058)
R axis (°)	68	(3, 95)	68	(5, 97)	69	(4, 97)	74	(12, 107)
	73	(14, 100)	71	(12, 97)	71	(9, 97)	70	(11, 95)
T axis (°)	43	(13, 64)	45	(15, 66)	47	(17, 69)	49	(14, 71)
	40	(0, 67)	41	(8, 67)	41	(5, 66)	41	(4, 68)

One of the key parameters to be measured from the ECG is the resting heart rate. In Table 2, it varies with age by decreasing steadily by about 15% from 87 to 73 bpm in males and from 89 to 76 bpm in females aged between 5 and 18 years. For the other variables considered, the trend reversed. The medians arise with age except for QTc which increased up to 11 years and then decreased slightly. Furthermore, the measures generally appeared higher among males except for QT interval from the age of 12 years onward, QTc interval from 8 years and P axis and R axis from 5 to 15 years. Finally, looking closely at the range of values for each parameter, the P wave and the QRS complex present a low dispersion between the 2nd and 98th percentiles unlike other parameters, such as the RR intervals. Overall, for each parameter and age group considered, the median values are comparable according to gender.

### ECG parameters comparison

After conducting a thorough review of the literature, we identified five studies to judge on whether our reference values were consistent with those already published or conversely they raised new questions. However, although very relevant, two were not retained in our analysis as the age groups considered were different from ours (6–9, 9–13 and 13–18 years for the first and 0–9 to 90–99 years for the second)^4,15^. Rijnbeek et al. conducted their study on 1912 healthy Dutch children aged 0–1 month to 12–16 years^5^. Palhares et al. analyzed 486,014 ECGs in Brazilian patients from 1–2 to 90 years and older^16^. For these two studies, median values and 2 and 98 percentile values are available. For the third study, the percentile values of interest were available for 2241 healthy Turkish children aged 0–1 day to 12–16 years^17^. The median values were not specified in this study, only mean values were given. To facilitate the reading of the discussion part, the main pieces of information related to these three studies are shown in Table 3.

**Table 3:** Main features of studies identified for the comparative analysis of ECG parameters.

Bibliographic reference	Study population characteristics			Main ECG parameters studied	Expression of data
	Origin	Age groups	Number		
Rijnbeek et al., 2001	Dutch	**11 days to 16 years** 0 to 1 month 1 to 3 months 3 to 6 months 6 to 12 months 1 to 3 years 3 to 5 years 5 to 8 years 8 to 12 years 12 to 16 years	1912	Heart rate P axis P duration PR interval QRS axis QRS duration QT_c_ interval	Median values 2nd and 98th percentiles values
Palhares et al., 2017	Brazilian	**1 to 90 years and older** 1 to 2 years 3 to 4 years 5 to 7 years 8 to 11 years 12 to 15 years 16 to 19 years 20 to 29 years … 80 to 89 years 90 years and older	486,014	Heart rate P frontal axis QRS frontal axis T frontal axis Overall P duration Overall PR interval Overall QRS duration QT interval QT_c_ Bazett	Median values 2nd and 98th percentiles values
Semizel et al., 2008	Turkish	**1 day to 16 years** 0 to 1 day 1 to 3 days 3 to 7 days 1 to 4 weeks 1 to 3 months 3 to 6 months 6 to 12 months 1 to 3 years 3 to 5 years 5 to 8 years 8 to 12 years 12 to 16 years	2241	Heart rate P wave duration PR interval QRS duration QT interval QT_c_ interval P wave axis T wave axis	Mean values 2nd and 98th percentiles values

### Heart rate

As seen previously, the heart rate is undeniably age-dependent and declines gradually in both genders until adulthood. This expected decrease with age observed in our study and in the three comparative studies^5,16,17^ is explained in particular by the volume of the heart that differs from birth to adulthood^18^. Overall, the extreme limits were quite close in all studies. Only the upper limits presented by the Turkish study were more pronounced for ages from 5 to 11 years (for instance, 133 bpm in males whereas the value was 110 bpm in our study)^17^.

### PR interval, P wave and QRS complex

In general, the compared studies showed an increase of these parameters with aging and the medians were most of the time similar by genders or slightly higher in males. For PR interval and QRS complex variables, the 2nd and 98th percentiles appeared in the same order of magnitude in all the studies considered. By contrast, the P wave duration was different in particular in the Turkish study. The upper and especially lower limits were markedly different from our study and those of the Dutch and Brazilian studies. For example, for children aged 5–7 years, the lower value was 40 ms for males compared to 66 ms in our study, 68 ms in the Brazilian study and 73 ms reported by the Dutch study. For the age group ”12–15″, the gap widened with 40 ms in males *versus* 72 ms in the Brazilian study and ours and 82 ms in the Dutch study.

### P axis

The median value for P axis tended to increase with time after a slight decline recorded for the age group “8–11” years. Nevertheless, a clear difference was noted for the lower limit. For example, our study and the Turkish study showed values of − 10° and − 7° in males of the category 5–7 age group, whereas the Dutch and the Brazilian studies distinguished with values of − 54° and − 22°, respectively. Moreover, while the 98th percentile values in our study and in the Dutch and Brazilian studies were always between 70° and 80° regardless of gender, those in the Turkish study were mostly > 100°.

### QTc

All QTc percentile values with age in our study were similar in the Brazilian and Turkish studies, but those in the Dutch study were lower. For example, for males aged 12–15 years, the QT_C_ was 407 ms (362–449) in the Dutch study, while it was 424 ms (383–464) and 419 ms (374–462) for our study and the Brazilian’, respectively.

### T axis

The T axis values in our study were similar as those in the Brazilian study. Differences were noted in the Turkish study for the children aged 12–14 years for the upper limit of up to 118° in males versus 69° in our study.

### Echocardiographic parameters

Echocardiographic parameters are presented in Table 4, from which two major points emerge.

**Table 4:** Echocardiographic parameters according to age and gender (males, upper row and females, lower row).

Echocardiographic parameters, median (2nd, 98th percentiles)	Age groups							
	5–7 years N = *1973*		8–11 years N = *4995*		12–15 years N = *4050*		16–18 years N = *1494*	
LVEDs (mm)	22	(18, 27)	24	(20, 29)	27	(22, 34)	29	(23, 35)
	21	(17, 26)	23	(19, 28)	26	(20, 32)	26	(21, 32)
LVEDd (mm)	37	(31, 43)	40	(33, 47)	46	(38, 53)	49	(41, 54)
	36	(29, 42)	38	(31, 46)	43	(36, 50)	45	(37, 51)
LVEF (%)	72	(62, 79)	71	(62, 79)	71	(62, 79)	71	(61, 78)
	72	(63, 79)	71	(62, 79)	71	(62, 79)	72	(63, 79)
LVPWd (mm)	6	(4, 9)	7	(5, 9)	8	(6, 10)	8	(6, 10)
	6	(4, 8)	6	(5, 9)	7	(5, 10)	7	(6, 10)
Left ventricle velocity (cm/sec)	94	(78, 110)	95	(79, 119)	96	(79, 115)	96	(78, 112)
	93	(77, 110)	95	(79, 115)	95	(76, 115)	95	(75, 115)
IVSd (mm)	6	(4, 8)	7	(5, 9)	8	(6, 10)	8	(6, 10)
	6	(4, 8)	6	(5, 9)	7	(5, 10)	8	(6, 10)
RVDd (mm)	13	(9, 18)	14	(9, 20)	15	(11, 25)	16	(11, 24)
	13	(9, 18)	13	(9, 20)	15	(11, 22)	15	(11, 23)
Mitral inflow (cm/sec)	96	(80, 118)	98	(81, 119)	100	(80, 119)	99	(80, 120)
	95	(80, 118)	98	(81, 118)	100	(80, 120)	99	(77, 120)
LAD (mm)	23	(19, 28)	25	(20, 31)	28	(23, 35)	29	(25, 36)
	22	(18, 27)	24	(19, 30)	27	(22, 34)	28	(24, 34)
AOD (mm)	20	(16, 24)	22	(17, 27)	25	(20, 31)	27	(22, 33)
	19	(16, 24)	21	(17, 26)	24	(20, 29)	25	(20, 30)
Tricuspid peak velocity (cm/sec)	68	(54, 89)	67	(52, 96)	68	(52, 92)	68	(54, 80)
	67	(53, 85)	67	(53, 95)	68	(53, 90)	69	(54, 84)
Pulmonary artery trunk diameter (mm)	14	(10, 18)	16	(11, 20)	17	(12, 22)	18	(13, 25)
	14	(10, 18)	16	(10, 20)	17	(12, 22)	18	(13, 23)
Pulmonary artery velocity (cm/sec)	90	(75, 107)	91	(75, 107)	93	(75, 107)	94	(75, 110)
	90	(75, 105)	91	(73, 107)	93	(74, 108)	92	(70, 105)

LVEDs, left ventricular diameter at end-systole; LVEDd, left ventricular diameter at end-diastole; LVEF, left ventricle ejection fraction; LVPWd, left ventricular posterior wall thickness at end-diastole; IVSd, interventricular septum thickness at end-diastole; RVDd, right ventricular diameter at end-diastole; LAD, left atrial diameter; AOD, aortic diameter.

Firstly, unlike ECG parameters, the median values of several echocardiographic parameters remain quite stable with age and the difference between 2nd and 98th percentiles is low. LVEF ranges from 72 to 71% in males and from 72 to 72% in females and tricuspid peak velocity goes from 68 to 68 cm/sec for males and from 67 to 69 cm/sec for females, aged between 5 and 18 years. In contrast, other data show a more substantial increase. This is the case for LVEDs, LVEDd, LAD and AOD, among others. For example, LVEDd varies from 37 to 49 mm for males and from 36 to 45 mm for females. Secondly, few differences are notable depending on gender. Indeed, similarly for ECG parameters, the median value of many echocardiographic parameters is similar in males and females according to age group.

The echocardiographic parameters indexed on BSA are listed in Table 5.

**Table 5:** Echocardiographic parameters indexed on BSA according to age and gender (males, upper row and females, lower row).

Echocardiographic parameters/BSA, mean ± SD	Age groups							
	5–7 years N = *1973*		8–11 years N = *4995*		12–15 years N = *4050*		16–18 years N = *1494*	
LVEDs/BSA (mm/m^2^)	23.23	± 2.93	20.76	± 2.87	17.74	± 2.33	16.18	± 1.67
	22.83	± 2.92	20.43	± 2.79	17.24	± 2.11	16.36	± 1.77
LVEDd/BSA (mm/m^2^)	38.95	± 4.33	34.62	± 4.30	29.55	± 3.48	26.90	± 2.33
	38.25	± 4.50	34.10	± 4.16	28.91	± 3.03	27.68	± 2.49
LVEF/BSA (%/m^2^)	74.99	± 10.11	62.02	± 10.08	45.97	± 7.74	39.09	± 4.87
	76.56	± 10.50	63.52	± 10.65	47.51	± 6.77	44.57	± 5.08
LVPWd/BSA (mm/m^2^)	6.32	± 1.21	5.72	± 1.08	5.04	± 0.87	4.62	± 0.65
	6.25	± 1.30	5.70	± 1.07	4.92	± 0.82	4.72	± 0.73
Left ventricle velocity/BSA (cm/sec/m^2^)	97.91	± 13.73	83.05	± 14.24	62.51	± 10.53	53.30	± 6.80
	99.68	± 14.55	84.80	± 14.53	63.89	± 9.84	59.18	± 7.84
IVSd/BSA (mm/m^2^)	6.41	± 1.17	5.78	± 1.05	5.10	± 0.85	4.68	± 0.63
	6.31	± 1.23	5.75	± 1.05	4.99	± 0.82	4.79	± 0.74
RVDd/BSA (mm/m^2^)	13.34	± 2.43	12.00	± 2.37	10.42	± 2.18	9.17	± 1.76
	13.43	± 2.51	12.04	± 2.38	10.31	± 1.98	9.76	± 1.84
Mitral inflow/BSA (cm/sec/m^2^)	101.75	± 15.49	85.82	± 14.53	64.76	± 11.53	55.10	± 7.79
	103.12	± 15.69	87.38	± 15.09	66.38	± 10.73	60.96	± 8.97
LAD/BSA (mm/m^2^)	23.92	± 2.92	21.53	± 2.84	18.36	± 2.35	16.52	± 1.70
	23.94	± 3.22	21.42	± 2.84	18.19	± 2.14	17.43	± 1.79
AOD/BSA (mm/m^2^)	20.88	± 2.67	19.05	± 2.67	16.30	± 2.22	15.07	± 1.64
	20.79	± 2.83	18.72	± 2.63	16.13	± 2.00	15.63	± 1.76
Tricuspid peak velocity/BSA (cm/sec/m^2^)	70.44	± 11.08	59.12	± 11.62	44.24	± 8.51	37.63	± 5.19
	71.75	± 12.14	60.41	± 12.11	45.28	± 7.39	42.70	± 6.00
Pulmonary artery trunk diameter/BSA (mm/m^2^)	15.13	± 2.95	13.42	± 2.70	10.92	± 2.24	9.94	± 2.08
	15.35	± 3.00	13.51	± 2.76	11.15	± 2.03	10.80	± 2.02
Pulmonary artery velocity/BSA (cm/sec/m^2^)	94.38	± 13.72	79.92	± 13.53	60.60	± 10.03	51.77	± 6.58
	96.55	± 14.12	81.42	± 14.24	61.88	± 9.41	57.05	± 7,41

*BSA,* body surface area*; SD,* standard deviation.

*LVEDs*, left ventricular diameter at end-systole; *LVEDd*, left ventricular diameter at end-diastole; *LVEF*, left ventricle ejection fraction; *LVPWd*, left ventricular posterior wall thickness at end-diastole; *IVSd*, interventricular septum thickness at end-diastole; *RVDd*, right ventricular diameter at end-diastole; *LAD*, left atrial diameter; *AOD*, aortic diameter.

Although Tables 4 and 5 are not totally comparable (median values in Table 4 and mean values in Table 5), we are able to highlight that the values for each parameter decline continuously as age increases. This decrease is very strong for several parameters, such as the left ventricle velocity/BSA for which the mean ranges from 97.91 to 53.30 cm/sec/m^2^ in males and 99.68 to 59.18 cm/sec/m^2^ in females aged between 5 and 18 years. As previously observed, the echocardiographic parameters appear quite close according to gender.

### Echocardiographic parameters comparison

The literature review highlighted four relevant articles discussing echocardiographic parameters, but methodological approaches were relatively different making comparison with our results complex. Bonatto et al*.* examined 595 healthy Brazilian children from 1 to 12 years old and presented results with percentile curves related to BSA and tables summarizing 3rd and 50th percentile values according to BSA^19^. The study of Majonga et al*.* involving 282 healthy children from Zimbabwe (sub-Saharan Africa) from 6 to 16 years old presented results with scatter plots against BSA including Z-scores values^20^. Kampmann et al*.* included 2036 healthy children from 1 day to 18 years old from central Europe^21^. The data have been grouped as centiles, charts with respect to BSA and tables with mean and SD values according to BSA and body weight. Finally, Overbeek et al*.* conducted a study on 747 healthy Dutch children from 1 day to 18 years old^22^. The results were shown through scatter plots in relation to body weight. All studies concluded that echocardiographic parameters were not significantly different according to gender, except for Majonga et al*.* (only for LVEDd parameter) and for Bonatto et al*.*

## Discussion

As previously stated, the primary objective of the study was to investigate a possible association between radiation exposure and cardiac arrhythmia in Russian children^1^. Therefore, we had the opportunity to acquire a number of electrocardiographic and cardiac ultrasound data on a large group of children. Such a magnitude of collected medical information on healthy children is a valuable input to enrich the information related to reference values of electrographic and cardiac ultrasound parameters, which remains rather scarce.

In this present study, we analyzed data collected through two simple and non-invasive medical examinations performed on 12,512 healthy children. First, an ECG which is a diagnostic tool of choice in the exploration of cardiac rhythms. Secondly, an echocardiography, an imaging technique that will allow, notably through anatomical and hemodynamic evaluation, identifying potential conditions leading to the occurrence of cardiac arrhythmia. In order to be as close as possible to the experimental conditions of previous studies addressing the issue, medical endpoints have been investigated according to gender and four age groups.

As for electrocardiographic parameters, the resting heart rate assessed in our study gradually slows down until adolescence. The median values range from 87 to 73 bpm for males and from 89 to 76 bpm for females. In contrast, P wave becomes steadily larger (the values range from 82 to 94 ms for males and from 82 to 92 ms for females) and QRS complex as well as the PR, QT and RR intervals longer, throughout the growth of children in both males and females. As an example, the QRS complex data go from 82 to 92 ms for males and from 78 to 86 ms for females. As for PR intervals, the values vary increasingly from 124 to 140 ms for males and from 124 to 136 ms for females. The measurements performed fully reflect the expected evolution of the paediatric electrocardiogram between 5 and 18 years of age.

As pointed out previously, differences exist between childhood and adulthood and they are notably explained by anatomical changes or tissue maturation^6,23^. Nevertheless, clarifications already mentioned in others publications are to be taken into account and contribute to the differences highlighted between studies. First of all, the equipment used is an important element to bear in mind and in particular its minimum sampling rate which, according to Dickinson et al., should be higher (minimum 500 or 1000 Hz)^24^. In our study, we used a device with a sampling rate of about 1200 Hz which was certainly more appropriate. However, our results were quite similar to those of the Palhares’ study. The number of individuals in a study is equally a parameter to be considered because a large number of individuals gives more reliable data. Finally, differences observed between studies may also be explained by racial disparities^25^. Masica’s study focused on 64 American males (33 Blacks and 31 Caucasians) aged 5 to 7 years^26^. Disparities including amplitude voltages or QRS complex duration were noted. Similarly, in a second study targeting 244 children in Saudi Arabia (124 Blacks and 120 Whites) 3 to 17 years old, the authors reported notable variations, in particular for the amplitudes of the R wave for children beyond 6 years of age^27^. Both studies recommend adjustment to race or the use of racially separate normal standards.

As for echocardiographic parameters, the values of LVEF, mitral inflow or tricuspid peak velocity assessed in our study remain rather stable with age and are similar between males and females. For instance, the LVEF values range from 72 to 71% for males and from 72 to 72% for females between 5 and 18 years. For other parameters like AOD, LVEDd or LAD, the evolution is more accentuated between 5 and 18 years in age. For example, data for AOD go from 20 to 27 mm for males and from 19 to 25 mm for females. The trend of these parameters is naturally expected due to the growth of the children.

Finally, all echocardiographic parameters indexed on BSA decrease steadily until adolescence. Some data have a very marked trend, as mitral inflow/BSA whose values go from 101.75 to 55.10 cm/sec/m^2^ for males and from 103.12 to 60.96 cm/sec/m^2^ for females.

In our study, we observed that anthropometric parameters represented characteristics to be considered since the echocardiographic values clearly could vary according to age classes. Furthermore, as all studies had been conducted in different places, the ethnic factor represents certainly a substantial element that could explain different results in the echocardiographic parameters study in children by gender^28^. Indeed, Majonga et al*.* who compiled the data from 36 studies on healthy children aged 5 to 21 years, differences were reported particularly for interventricular septal thickness among Black African, Indian, German and US American children^29^.

In conclusion, taking advantage of data from the large cross-sectional study on the health consequences of the Chernobyl nuclear accident conducted on Russian children, sharing with the scientific community the numerous electrocardiographic and cardiac ultrasound data collected seems relevant in order to shed new light on the issue of the reference values of these parameters in children. Obviously, it is not easy to reach a consensus as scientific approaches may be different and especially for echocardiography. In contrast, apart from especially the equipment used and the size of the study population, the anthropometric and racial/ethnic factors constitute key elements to be taken into account in the study of ECG and echocardiographic parameters in children and may certainly explain the differences observed with other studies.
